# Kinetics of humoral and cellular immune responses 5 months post-COVID-19 booster dose by immune response groups at the peak immunity phase: An observational historical cohort study using the Fukushima vaccination community survey

**DOI:** 10.1016/j.jvacx.2024.100553

**Published:** 2024-09-12

**Authors:** Yurie Kobashi, Takeshi Kawamura, Yuzo Shimazu, Yudai Kaneko, Yoshitaka Nishikawa, Akira Sugiyama, Yuta Tani, Aya Nakayama, Makoto Yoshida, Tianchen Zho, Chika Yamamoto, Hiroaki Saito, Morihito Takita, Masatoshi Wakui, Tatsuhiko Kodama, Masaharu Tsubokura

**Affiliations:** a Department of Radiation Health Management, Fukushima Medical University School of Medicine, Fukushima City, Fukushima, Japan; b Department of General Internal Medicine, Hirata Central Hospital, Hirata, Ishikawa District, Fukushima, Japan; c Isotope Science Center, The University of Tokyo, Tokyo, Japan; d Laboratory for Systems Biology and Medicine, Research Center for Advanced Science and Technology, The University of Tokyo, Tokyo, Japan; e Medical & Biological Laboratories Co., Ltd, Tokyo, Japan; f Medical Governance Research Institute, Minato-ku, Tokyo, Japan; g Department of Laboratory Medicine, Keio University School of Medicine, Tokyo, Japan; h General Incorporated Association for Comprehensive Disaster Health Management Research Institute, Japan

**Keywords:** COVID-19, SARS-CoV-2, Booster dose vaccine effectiveness, Humoral immunity, Cellular immunity

## Abstract

**Background:**

Understanding the waning of immunity after booster vaccinations is important to identify which immune-low populations should be prioritized.

**Methods:**

We investigated longitudinal cellular and humoral immunity after the third vaccine dose in both high- and low-cellular and humoral immunity groups at the peak immunity phase after the booster vaccination in a large community-based cohort. Blood samples were collected from 1045 participants at peak (T1: median 54 days post-third dose) and decay (T2: median 145 days post-third dose) phases to assess IgG(S), neutralizing activity, and ELISpot responses. Participants were categorized into high/low ELISpot/IgG(S) groups at T1. Cellular and humoral responses were tracked for approximately five months after the third vaccination.

**Results:**

In total, 983 participants were included in the cohort. IgG(S) geometric mean fold change between timepoints revealed greater waning in the >79 years age group (T2/T1 fold change: 0.27) and higher IgG(S) fold change in the low-ELISpot group at T1 (T2/T1 fold change: 0.32–0.33) than in the other groups, although ELISpot geometric mean remained stable.

**Conclusions:**

Antibody level of those who did not respond well to third dose vaccination waned rapidly than those who responded well. Evidence-based vaccine strategies are essential in preventing potential health issues caused by vaccines, including side-effects.

## Introduction

Vaccination is a robust approach against infectious diseases; it emerged as the most effective strategy worldwide against the recent SARS-CoV-2 pandemic, which has caused more than 6.5 million deaths to date. However, to overcome COVID-19, many issues, such as disparities in access to vaccinations [1], hesitancy against vaccination [2], short- and long-term secondary effects of the disease [3], [4], loss of vaccine efficacy against transmissible SARS-CoV-2 variants [5], and the waning of immunity over time [6], must be addressed. All these issues should be taken into account while developing an appropriate vaccine strategy in the future. Understanding the waning of immunity after booster vaccinations is important to identify which immune-low populations should be prioritized. To address this, immunity in SARS-CoV-2 infection-naive populations should be evaluated after they have received a booster dose, to identify the most advantageous vaccination approach and assess herd immunity.

Various studies have been conducted to evaluate immunity in SARS-CoV-2 infection-naive populations. Such evaluations are important, as cellular immunity is associated with the prevention of severe diseases, which is the aim of the current booster vaccinations, in addition to infection control. Previous studies have reported that low cellular immunity is associated with various factors and populations, including short intervals between vaccinations [7], patients with solid cancers [8], [9], patients with hematopoietic malignancies [10], [11], [12], transplant recipients [13], [14], [15], [16], vaccination types [17], patients with common variable immunodeficiency [18], [19], hematopoietic stem cell transplant recipients [20], patients receiving anticancer treatment [21], [22], [23], uninfected populations [24], patients with immune-mediated inflammatory diseases [25], those receiving immunomodulatory therapy [26], [27], and patients undergoing hemodialysis [28], [29]. However, booster doses can generate sufficient cellular immunity responses, even among low-immunity populations [11], [30], [31], [32], [33], [34], [35], [36], [37]. Few studies have reported on cellular and humoral longitudinal immunity in large community-based SARS-CoV-2 infection-naïve cohorts after the third vaccine dose.

In Japan, more than 33 million patients with SARS-CoV-2 infection and more than 74,000 related deaths have been reported as of May 2023, when notifiable disease surveillance had closed. [38]. To investigate immunity associated with COVID-19, medical institutes and the municipalities of the Hirata Village, Soma City, and Minamisoma City, which experienced a triple disaster after the Great East Japan Earthquake, and Fukushima Medical University conducted the Fukushima Vaccination Community Survey (FVCS) with support from the Japan Agency for Medical Research and Development (AMED). The findings of FVCS were shared with the wider society and community [2], [3], [39], [40], [41], [42], which enhanced the knowledge about COVID-19 immunity and the SARS-CoV-2 vaccine. This growing understanding has created ideal conditions for conducting a community-based longitudinal cohort study on cellular and humoral immunity following the third COVID-19 vaccination.

The objective of this study was to identify preoptimized populations suitable for additional booster doses, aiming to prevent the occurrence of severe disease. To this end, we investigated the longitudinal cellular and humoral immunity after the administration of the third vaccine dose in both high- and low-cellular and humoral immunity groups during the peak immunity phase after the booster vaccination in the infection-naive cohort.

## Methods

### Study design and participants

This was an observational historical cohort study that was conducted as part of the FVCS. The study was approved by the ethics committees of Hirata Central Hospital (number 2021-0611-1) and Fukushima Medical University (number 2021-116). All participants provided written informed consent. This study conforms to The Code of Ethics of the World Medical Association (Declaration of Helsinki). The authors had access to information that could identify individual participants during and after data collection.

Health professionals from the Seireikai group and associated facility who dwell mainly in the Hirata village participated in this study. Location details are reported elsewhere [3]. To evaluate immunology after the third dose of the SARS-CoV-2 vaccine, we included participants who had already received two doses of BNT162b and a third dose of BNT162b (Pfizer) or mRNA1273 (Moderna) to evaluate their cellular and serological immune responses. Participants who had completed their third vaccination (booster vaccination) had their blood sampled during the peak phase (T1: median of the day from the third vaccine was 54 days) and decay phase (T2: median of the day from the third vaccine was 145 days). We excluded participants who received their third doses between T1 and T2 or who were infected by T2 and who could not obtain the appropriate cellular immunology results, as per the official procedure guidelines (Supplementary Fig. 1). Blood sampling in the peak (T1) and decay phases (T2) was conducted by health care staff from the Seireikai group in March and July 2022, respectively. A paper-based questionnaire was also conducted to obtain relevant data, including social demographics, adverse reactions, comorbidities, daily medication, and vaccination information.

### Cell immune response assays

We evaluated cellular immune responses using ELISpot with T-spot COVID (Oxford Immunotec; UK). Blood samples were transferred from the hospital to the LSI Medience Corporation within the blood sampling day to conduct ELISpot testing; subsequently, all tests were performed by the LSI Medience Corporation as per the official guidelines. The ELISpot target antigen was the Spike protein; however, we could not obtain detailed information on the spike protein peptide pool used for T-spot(S).

Peripheral blood mononuclear cells (PBMC) were sampled; the number of PBMC was adjusted to 2.5 × 10^5^ cells/100 μL, and 250,000 ± 50,000 cells were seeded per well. PBMC and the ELISpot target antigen were added to the wells with the labelled antibody. After removing unnecessary cells, interferon-gamma (IFN-γ)-generating effector T cells were counted as spots on the wells. The results of positive and negative control wells were compared. According to the official guidelines, the spots on the wells were counted up to 50L: >50 spots was shown as “50 and over spots”; >7 spots was judged to be reactive; 5–7 spots were judged to be borderline; and <5 spots was judged to be not reactive.

### Serological assay

IgG(S) and NAb were used to evaluate the serological immunity. All serological assays were performed using the CLIA assay with iFlash 3000 (YHLO Biotech, Shenzhen, China) and iFlash-2019-nCoV series (YHLO Biotech, Shenzhen, China) as reagents in Tokyo University between March and August 2022. The correlations between IgG(S) and NAb have been reported previously [3].

For the NAb assay, incubation was performed to cause a reaction between the NAb and Receptor Binding Domain (RBD) antigen-coated paramagnetic microparticles after washing out the non-reacted materials. Subsequently, the resulting chemiluminescent reaction was quantified as relative light units using a calibration curve. To distinguish between vaccinated and non-vaccinated participants, the official cut-off values were 10 arbitrary units per milliliter (AU/mL) for IgG(S) and NAb; however, this cut-off value might not be useful to evaluate outcomes within the vaccinated population. Because the official measuring range for NAb accuracy was set at 800, any value greater than 800 was treated as a value of 800. The official measuring range for IgG(S) accuracy was set at 3500; however, we handled data over 3500 because the accuracy of high values was established by comparing the NAb reported previously [3]. IgG(S) was generally sufficiently stable to evaluate the high antibody titer compared to NAb.

### Outcomes

The coprimary outcomes were longitudinal immunogenicity approximately five months after the third dose, with each low and stable responder group at the peak phase after the third dose. Immunogenicity was assessed using different assays including hormonal immunity with IgG(S) and NAb, and cellular immunity with ELISpot. Groups were generated first based on the immunity at the peak phase (T1), and the lower 25th quantile of the ELISpot (<5, equally with not reactive in the official guidelines; n = 218) and IgG(S) (<1404.5; n = 245) were used for grouping. We defined Group 1 as ELISpot high and IgG(S) high, Group 2 as ELISpot high and IgG(S) low, Group 3 as ELISpot low and IgG(S) high, and Group 4 as ELISpot low and IgG(S) low. Group 4 was expected to be a low responder after the third dose (Fig. 2A). We tracked the cellular and humoral responses to the decay phase, approximately 5 months after the third vaccination, in each group.

### Statistical analysis

This study aimed to investigate the longitudinal cellular immunity response and antibody kinetics in each low and stable responder group after the third vaccine dose. To evaluate the low responders after the third dose, we defined 4 groups by their humoral and cellular immunity at the peak phase (T1). We reported the age distributions for each group.

The geometric mean of the neutralizing activity and IgG antibody and ELISpot for each age group were reported at T1 and T2. The fold changes between the two timepoints were calculated using age groups. In addition, the geometric mean of ELISpot and antibody titers for each immune fragility group (Groups 1–4) were reported at T1 and T2. The fold changes between the two timepoints were calculated using the immune fragility group. An official evaluation of ELISpot at T2 (reactive, borderline, not reactive) showed all immune groups at T1. The antibody titers at T2 showed the reactivity of ELISpot at T2 in immune Groups 1–4.

The distribution of neutralizing activity, IgG antibody titers, and ELISpot at T1 (March 2022) and T2 (June 2022) is shown using a histogram (Supplementary Fig. 1). The characteristics of all participants were reported using the four immune level groups at T1 (peak phase). Chi-square tests were used to analyze the categorical variables, and ANOVA was used for the continuous variables. Logistic regression analysis was performed to identify the factor associated with the lower IgG(S) group and ELISpot group at T2. The dependent variables were defined as (1) the group of the lower 33rd quantile of IgG (S) at T2, (2) the group of the lower 33rd quantile ELISpot at T2, and (3) the group of the lower 33rd quantile of both IgG(S) and ELISpot; hence, three logistic regression models were performed. The independent variable was selected using Akaike's information criterion and the Bayesian information criterion and based on previous studies reporting each model. In the figures, IgG(S) antibody titers over 5000 are shown as 5000, and NAbs over 800 are shown as 800. Statistical analyses were performed with STATA IC (Lightstone, TX, USA, version 15).

## Results

**Fig. 1:**
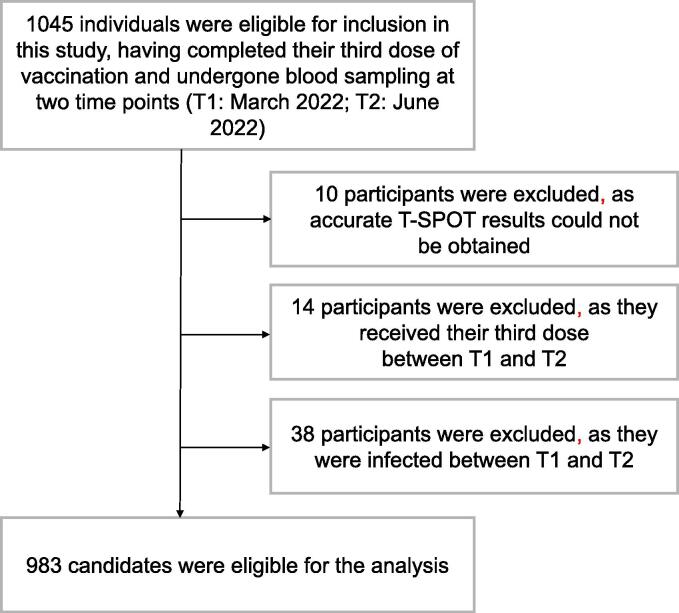
Participant selection criteria. Participants were included in this study if they had received two doses of BNT162b and a third dose of BNT162b (Pfizer) or mRNA1273 (Moderna) and if they had their blood sampled during the peak phase (T1: median of day from third vaccine was 54 days) and decay phase (T2: median of day from third vaccine was 145 days). Participants who received their third doses between T1 and T2 or who were infected by T2 and who could not obtain the appropriate cellular immunology results were excluded.

Of the 1045 participants who were administered a third vaccination and who provided two blood samples for the ELISpot and serological assays, 983 were determined to be eligible for inclusion in the present study (Fig. 1). All participants were administered two doses of BNT162b2; 356 individuals (36.2 %) received mRNA-1273 (Moderna), while the others received BNT162b as their third dose. The median interval between the second and third doses for the entire cohort was 230 days (interquartile range (IQR): 217–238, Table 1).

**Fig. 2:**
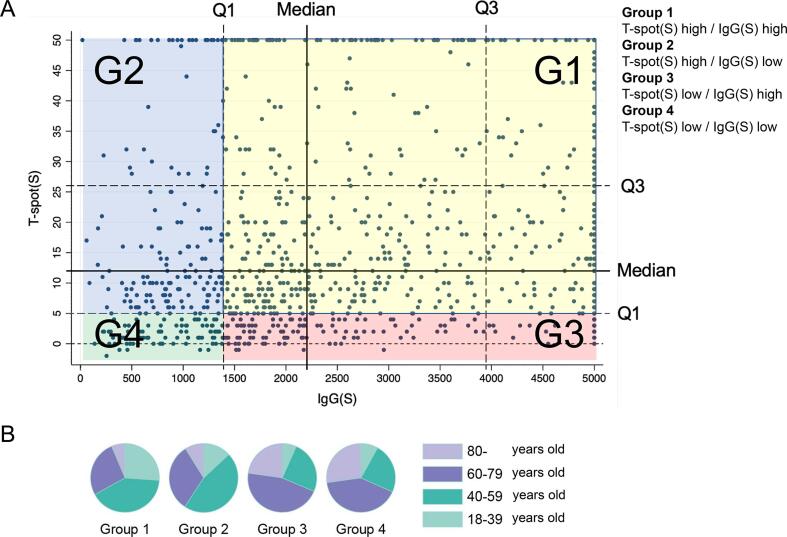
Immune level groups at T1 and age distribution. (A) Groups were designated based on the immunity at the peak phase (T1), the lower 25th quantile of ELISpot (<5, equally with not reactive in the official guidelines; n = 218), and IgG(S) (<1404.5; n = 245). Group 1 was defined as ELISpot high and IgG(S) high, Group 2 was ELISpot high and IgG(S) low, Group 3 was ELISpot low and IgG(S) high, and Group 4 was ELISpot low and IgG(S) low. IgG(S) antibody titers over 5000 are shown as 5000 in the figure and NAb over 800 are shown as 800 in the figure. (B) Age distribution in each cohort group (Groups 1–4).

**Table 1:** Characteristics of the study participants (N = 983).

	n (%)
Age, years (median [IQR])	56 [42–68]
Sex, female	659 (67.0)
Interval between first blood sampling day (T1) and 3rd vaccine (median [IQR])	54 [29–65]
Interval between second blood sampling day (T2) and 3rd vaccine (median [IQR])	145 [124–158]
Interval day between 1st and 2nd vaccines (median [IQR])	21 [21–21]
Interval days between 2nd and 3rd vaccines (median [IQR])	230 [217–238]

Type of third dose vaccination	
mRNA-1273	356 (36.2)
BENT162b	627 (63.8)

Adverse reaction	
Local pain (n = 978)	601 (61.5)
Fever under 37.5℃ (n = 978)	129 (13.2)
Fever over 37.5℃ (n = 978)	259 (26.5)
Fatigue (n = 978)	440 (45.0)
Headache (n = 977)	268 (27.4)
Joint pain (n = 978)	296 (30.3)
Diarrhea (n = 976)	27 (2.8)
Nausea (n = 978)	43 (4.4)
Dizziness (n = 977)	44 (4.5)
Others (n = 974)	66 (6.8)
Smoking habit (n = 964)	174 (18.1)
Alcohol consumption (n = 952)	379 (39.8)

Daily medicine	
Steroid (n = 961)	26 (2.7)
Immunosuppressants (n = 960)	14 (1.5)
Biologics (n = 958)	5 (0.5)

Comorbidity	
Hypertension (n = 981)	258 (26.3)
Diabetes (n = 981)	78 (8.0)
Dyslipidemia (n = 981)	87 (8.9)

BMI (n = 867)	
18.5–25	554 (63.9)
<18.5	55 (6.3)
25–30	196 (22.6)
<30	62 (7.2)

Total number of each variable was changed because of the missing data from the questionnaire survey.

The geometric means for each assay were analyzed according to age groups. Geometric means for each assay were notably elevated in the “under 40 years of age” group. An assessment of fold changes in the levels of immunoglobulin G against Spike 1 protein [IgG(S)] and neutralizing activity (Nab) between the two time points revealed pronounced decline in antibody titers, particularly evident in the “>79 years of age” group. Conversely, the fold changes in ELISpot between the two time points indicated a significant decline in ELISpot reactivity within the younger group (Table 2).

**Table 2:** T-spot(S) for each age group (N = 983).

	T1 (March 2022)	T2 (June 2022)	T2/T1
Neutralizing activity			
≤39	771.7 (748.7–795.4)	627.8 (584.0–675.0)	0.81
40–59	724.6 (698.5–751.7)	522.2 (483.5–564.0)	0.72
60–79	732.3 (699.7–766.5)	531.9 (492.3–574.6)	0.73
≥80	569.9 (490.5–662.1)	266.7 (206.6–344.3)	0.47
Total	717.5 (698.7–736.9)	507.0 (481.9–533.4)	0.71
IgG(S)			
≤39	2812.2 (2540.1–3113.4)	1123.2 (997.0–1265.3)	0.40
40–59	2164.2 (1992.8–2350.4)	844.0 (770.2–924.9)	0.39
60–79	2110.9 (1937.1–2300.4)	800.3 (727.1–881.0)	0.38
≥80	1689.1 (1393.4–2047.5)	451.6 (359.7–567.0)	0.27
Total	2202.8 (2093.4–2317.9)	821.7 (774.8–871.5)	0.37
T-spot(S)			
≤39	16.9 (15.0–19.0)	13.3 (11.5–15.4)	0.79
40–59	14.4 (13.0–15.9)	10.8 (9.7–12.1)	0.75
60–79	9.0 (7.9–10.2)	8.2 (7.2–9.3)	0.91
≥80	5.2 (4.2–6.5)	5.1 (4.0–6.5)	0.98
Total	11.6 (10.9–12.5)	9.7 (9.0–10.4)	0.84

Numbers of participants in each age group, under 40, 40–59, 60–70, and over 79 years, were 195, 375, 308, and 105, respectively.

The distribution of the individual results based on the cellular and hormonal immune response groupings at the peak phase (T1) is shown in Fig. 2. We defined Group 1 as ELISpot high and IgG(S) high, Group 2 as ELISpot high and IgG(S) low, Group 3 as ELISpot low and IgG(S) high, and Group 4 as ELISpot low and IgG(S) low. In total, 605 participants were included in Group 1, 160 in Group 2, 133 in Group 3, and 85 in Group 4, which was the least immune group owing to the presence of low humoral aa well as cellular immune responses.

The age-group proportions for all four immune groups are shown in Fig. 2B. The median ages of participants in Groups 1, 2, 3, and 4 were 52, 54.5, 67, and 66 years, respectively (Supplementary Table 1). The number of participants by age groups in different immune groups at T1 is shown in Supplementary Table 2. Notably, the proportion of individuals aged >80 years was 6.3 % in Group 1, 8.8 % in Group 2, 22.6 % in Group 3, and 27.1 % in Group 4.

Subsequently, the geometric means for each assay were discussed for the 1–4 immune response group at T1. The fold change in IgG(S) was small in Groups 3 and 4, although the IgG(S) antibody titer was higher in Group 3 than in Group 2 at T1. No decrease in the ELISpot geometry was observed between T1 and T2 among Groups 3 and 4, which exhibited low ELISpot values at T1 (Table 3).

**Table 3:** Geometric mean (95 % CI) of neutralizing activity, IgG antibody titers, and T-spot (S) for each immune group (N = 983).

	T1 (March 2022)	T2 (June 2022)	T2/T1
Neutralizing activity			
Group 1	797.5 (795.8–798.8)	711.7 (695.9–727.8)	0.89
Group 2	555.7 (497.4–620.8)	223.6 (192.8–259.5)	0.40
Group 3	796.2 (792.3–800.1)	605.7 (558.5–656.9)	0.76
Group 4	465.6 (386.5–560.8)	160.5 (122.5–210.2)	0.35
IgG(S)			
Group 1	3253.8 (3118.7–3394.7)	1265.3(1197.5–1336.9)	0.39
Group 2	787.0 (716.2–864.8)	299.8 (271.7–330.9)	0.38
Group 3	2601.2 (2404.13–2814.5)	856.8 (759.4–966.7)	0.33
Group 4	733.9 (650.0–828.5)	237.8 (202.8–278.9)	0.32
T-spot(S)			
Group 1	17.7 (16.7–18.8)	13.4 (12.3–14.5)	0.76
Group 2	14.8 (13.2–16.5)	11.0 (9.4–12.9)	0.74
Group 3	2.3 (2.1–2.5)	3.2 (2.8–3.7)	1.39
Group 4	2.0 (1.7–2.2)	2.7 (2.2–3.3)	1.35

**Fig. 3:**
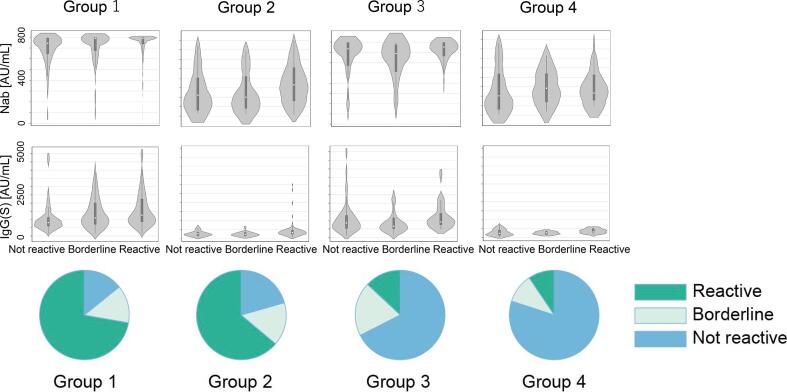
T-spot(S), Nab, and IgG(S) results at T2 (June 2022) by the immune level group at T1 (March 2022, Fig. 2). Results of the ELISpot at T2 (June 2022) in the immune 1–4 group at T1 (March 2022; see Fig. 2). The antibody titers at T2 indicated by the reactivity of ELISpot at T2 in immune Groups 1–4. Pattern of neutralizing antibodies (NAb), IgG (S), and T-spot at T2 phase among individuals who received a third dose booster. Individuals were divided into groups 1 to 4 based on the immune status of IgG(S) and T-spot at T1. Violin plots show the median level and interquartile range of NAb and IgG (S) according to non-reactive, borderline, and reactive immune status. The pie chart illustrates the proportion of the T-spot reactivity status at T2. IgG(S) antibody titers over 5000 are shown as 5000; NAb over 800 is shown as 800.

In total, 444 individuals (73.4 %) maintained their ELISpot reactivity at T2 in Group 1, while 102 individuals (63.8 %) were ELISpot reactive in Group 2. In total, 45 (33.8 %) and 19 individuals (22.4 %) were reactive to ELISpot again at T2 in Groups 3 and 4, respectively. The antibody titers were slightly high among participants who were ELISpot reactive at T2 in each immune Group (Fig. 3).

The distribution of Nab and IgG antibody titers was markedly altered between T1 and T2; however, the distribution of ELISpot was not considerably altered between T1 and T2 (Supplementary Fig. 1). The proportion of individuals who were vaccinated with mRNA-1273 as a third dose was low in Group 2 (Supplementary Table 1). Significant variables associated with the group with the lower 1/3 IgG (S) and T-spot (S) were aging, the BNT162b vaccine, no whole adverse reaction, smoking habit, and daily use of immunosuppressants (Supplementary Table 3).

## Discussion

To develop a comprehensive COVID-19 vaccination strategy, it is crucial to identify individuals with diminished cellular and humoral immune responses following the administration of booster doses among infection-naive populations.

In this study, the most significant decrease in longitudinal humoral immunity was observed in Group 4, characterized by low cellular and humoral responses at the peak phase after the third vaccination. However, no further decline in cellular immunity was noted. The fold changes in the levels of IgG antibodies were 0.39 and 0.38 in Groups 1 and 2 (indicating high cellular immune response at T1), respectively, compared to 0.33 and 0.32 in Groups 3 and 4 (reflecting low cellular immune response at T1), respectively. Previous cross-sectional studies have shown a weak association between cellular and humoral immunity [43]. Our findings suggest a connection between cellular immunity at the peak phase and longitudinal antibody kinetics. Thus, it is essential to utilize diverse immune kinetics assays for accurately identifying individuals at risk of severe diseases post-booster dose.

Antibody titers steadily decreased from the peak phase after the third dose for five months among all groups; cellular immunity decreased only among the groups with high cellular immunity at the peak phase after the third vaccination and not among those with low cellular immunity at the peak phase. Fold changes in the IgG antibody were 0.37 among the total population; however, the fold changes in the ELISpot values were 0.74 and 0.72 in Groups 1 and 2 (higher cellular immune response at T1), respectively, while those in Groups 3 and 4 were 1.27 and 1.30 (low cellular immune response at T1), respectively. The second booster vaccination aims to partly prevent an increase in the risk of developing severe COVID-19 [44]. A rapid decrease in antibody titers was consistent with the fact that the ability to prevent infection decreased from the day the booster was received. A portion of the population was not reactive to ELISpot at the peak phase; however, this was not consistent with the fact that severe disease was efficiently prevented across the population by the booster dose. A previous study has reported that low humoral and cellular immune responses early after breakthrough infection contributed to severe COVID-19 [45]. Yet, further studies are required to identify how cellular and humoral immunity is associated with the prevention of severe disease. In addition, further detailed assays among the groups with low cellular immunity and prospective cohort studies regarding the incidence of infection and occurrence of severe disease in low- and high-immunity groups are required to accurately identify the vulnerable population who will require the administration of repeated booster doses.

Aging exhibits a stronger correlation with low cellular immunity than with low humoral immunity at the peak phase. In Group 2, comprising individuals with low humoral immunity, 40.6 % were aged ≥60 years, whereas in Group 3, consisting of individuals with low cellular immunity, this proportion rose to 68.4 %. Similarly, in Group 4, characterized by low levels in both antibody and ELISpot, 68.2 % were aged ≥60 years. Aging is a recognized determinant of disease severity [46]. The highest proportion of aging participants was observed in the group displaying deficits in both cellular and humoral immunity. This underscores the importance of employing diverse immunity assays for a comprehensive assessment of post-booster vaccinations.

Assays to assess immunity among the infection-naive population after the booster vaccination are lacking. In general, cellular immunity reportedly waned after the peak phase of the booster dose [47]. In addition, a previous study showed that 99 % of healthy individuals were IFN-γ positive [48]. However, the geometric mean of the ELISpot values increased during the decay phase of the booster dose in Groups 3 and 4, representing individuals with low cellular immunity at the peak phase. These results suggest that the accuracy of the present ELISpot method is uncertain with regard to the non-reactive ELISpot group. Additionally, the results of the cellular immune assay may not be sufficient to discuss protection against infection and individual disease severity. Moreover, evaluating the neutralizing activity at high titer levels is challenging, and a correlation with IgG(S) could not be obtained above 800. In general, the investment for immune assays for COVID-19 might be difficult to prioritize compared to that for vaccine development and other profitable sectors, in terms of marketing. However, it is essential to develop evidence-based vaccine strategies to manage epidemic and pandemic of infectious disease like as COVID-19. Various challenges remain in assessing the immunity of naïve populations after the administration of booster doses; reliable testing methods should be established, and resource investment should be considered in this area.

Several limitations should be considered when interpreting the results of this study. First, ELISpot measured the IFN-γ levels in effector T cells and other cells, making it difficult to discuss the cellular immune response. Further cellular testing, such as cell specified flow cytometry, are required to identify low cellular immune responder. Second, the cohort was not standardized, as it consisted of 67 % female participants and individuals from diverse social and clinical backgrounds. Third, we did not perform the humoral assay across a range of dilutions. Fourth, we did not assess high values of cellular immunity. In the future, additional assays, such as activation-induced marker assays, should be considered, Fifth, we did not have baseline samples for T-spot(S) and thus could not clarify whether cellular immunity was induced by vaccination in Groups 3 and 4. Additional assays for cellular immunity are required to investigate vaccination-induced immunity in Groups 3 and 4. Sixth, we could not obtain detailed information on the spike protein peptide pool used for T-spot(S). We requested the manufacturer several times to provide the information about peptide pool; however, we could not obtain these details. Before starting the survey, we should check whether we could obtain this information on antigen. Lastly, while eight participants were IgG(N) positive at T2, they did not test positive for SARS-CoV-2 infection in a PCR test. Thus, we included these participants in analysis. Despite these limitations, the present study is the first to survey the humoral and cellular immune responses for 5 months after the administration of the third dose of the COVID-19 vaccine in a large cohort in Japan.

Following the peak phase, low humoral and cellular immune responses were observed at 5 months post-administration of the COVID-19 booster vaccine. The decline in longitudinal antibody levels was more pronounced among individuals with low humoral and cellular immune responses than among those with higher immune responses; however, no further decrease in cellular immunity was noted. Implementing evidence-based vaccine strategies is crucial for mitigating potential vaccine-related health issues. Numerous challenges persist in assessing populations with low cellular immunity; addressing these challenges necessitates the development of reliable testing methods and allocation of resources to advance this area of research.

## Funding

The financial support for this research was divided based on distinct components of the study. Data acquisition was principally funded by the 10.13039/100009619AMED (Japan Agency for Medical Research and Development) under the funding title “Development of Vaccines for the Novel Coronavirus Disease,” with grant no. JP21nf0101638. The data analysis and interpretation phase was primarily supported by Moderna Inc. It is important to note that the two funding bodies, AMED and Moderna Inc., are not related to each other in any capacity. The content of this study does not necessarily represent the official views of either funding body. The opinions, findings, conclusions, or recommendations expressed herein are solely those of the authors and do not reflect the perspectives of the aforementioned funding agencies. Additionally, this work was supported by JSPS KAKENHI Grant Number 23H00503, and by Medical & Biological Laboratories Co., Ltd. and Shenzhen YHLO Biotech Co., Ltd., the distributor and manufacturer of the antibody measurement system (iFlash 3000). This research was also supported by grants from the Kowa Co. and Research Center for Advanced Science and Technology in the 10.13039/501100004721University of Tokyo.

## Author contributions

Conception of the work: YK, YS, TKa, YN, TKo, MTs.

Data collection: YK, YS, YT, MY, ZH, CY, MTs.

Examinations: TKa, YK, AS, AN, TKo.

Data analysis and interpretation: YK, MTs.

Data curation: YK, MTs.

Drafting of the article: YK.

Critical revision of the article: MTs.

Final approval of the version to be published: all authors.

## CRediT authorship contribution statement

**Yurie Kobashi:** Writing – original draft, Formal analysis, Conceptualization. **Takeshi Kawamura:** Resources, Investigation. **Yuzo Shimazu:** Writing – review & editing. **Yudai Kaneko:** Investigation. **Yoshitaka Nishikawa:** Writing – review & editing. **Akira Sugiyama:** Writing – review & editing. **Yuta Tani:** Writing – review & editing. **Aya Nakayama:** Investigation. **Makoto Yoshida:** Writing – review & editing. **Tianchen Zho:** Writing – review & editing. **Chika Yamamoto:** Writing – review & editing. **Hiroaki Saito:** Writing – review & editing. **Morihito Takita:** Writing – review & editing. **Masatoshi Wakui:** Writing – review & editing. **Tatsuhiko Kodama:** Writing – review & editing, Supervision. **Masaharu Tsubokura:** Writing – review & editing, Supervision, Methodology, Funding acquisition.

## Declaration of competing interest

The authors declare the following financial interests/personal relationships which may be considered as potential competing interests: Kaneko is employed by Medical & Biological Laboratories, Co. (MBL, Tokyo, Japan). MBL imported the testing material used in this research. Kaneko participated in the testing process but was not involved in the research design and analysis. Kobashi and Tsubokura received a grant from Pfizer Health Research Foundation for research unrelated to this work.

## Data Availability

The datasets generated for this study are not available publicity but are available for direct access on reasonable request from the corresponding author under the permission of Fukushima Medical University.

## References

[1] Williams N. (2021). Assessment of racial and ethnic disparities in access to COVID-19 vaccination sites in Brooklyn, NY. JAMA Netw Open.

[2] Yoshida M. (2022). Factors associated with COVID-19 vaccine booster hesitancy: a retrospective cohort study, Fukushima vaccination community survey. Vaccines (Basel).

[3] Kobashi Y. (2022). Factors associated with anti-severe acute respiratory syndrome coronavirus 2 (SARS-CoV-2) spike protein antibody titer and neutralizing activity among healthcare workers following vaccination with the BNT162b2 vaccine. PLoS One.

[4] Riad A. (2021). COVID-19 vaccines safety tracking (CoVaST): protocol of a multi-center prospective cohort study for active surveillance of COVID-19 vaccines’ side effects. Int J Environ Res Public Health.

[5] Diamond M. (2021). SARS-CoV-2 variants show resistance to neutralization by many monoclonal and serum-derived polyclonal antibodies. Res Sq.

[6] Goldberg Y. (2021). Waning immunity after the BNT162b2 vaccine in Israel. N Engl J Med.

[7] Flaxman A. (2021). Reactogenicity and immunogenicity after a late second dose or a third dose of ChAdOx1 nCoV-19 in the UK: a substudy of two randomised controlled trials (COV001 and COV002). Lancet.

[8] Shroff R.T. (2021). Immune responses to two and three doses of the BNT162b2 mRNA vaccine in adults with solid tumors. Nat Med.

[9] Shroff RT et al. Immune responses to COVID-19 mRNA vaccines in patients with solid tumors on active, immunosuppressive cancer therapy. medRxiv; 2021.

[10] Re D. (2022). Humoral and cellular responses after a third dose of SARS-CoV-2 BNT162b2 vaccine in patients with lymphoid malignancies. Nat. Commun..

[11] Schubert L. (2022). Immunogenicity of COVID-19 vaccinations in hematological patients: 6-month follow-up and evaluation of a 3rd vaccination. Cancers (Basel).

[12] Morawska M. (2022). Reasons and consequences of COVID-19 vaccine failure in patients with chronic lymphocytic leukemia. Eur J Haematol.

[13] Reindl-Schwaighofer R. (2022). Comparison of SARS-CoV-2 antibody response 4 weeks after homologous vs heterologous third vaccine dose in kidney transplant recipients: a randomized clinical trial. JAMA Intern Med.

[14] Yahav D. (2022). Immune response to third dose BNT162b2 COVID-19 vaccine among kidney transplant recipients-a prospective study. Transpl Int.

[15] Meshram H.S. (2022). Humoral and cellular response of COVID-19 vaccine among solid organ transplant recipients: a systematic review and meta-analysis. Transpl Infect Dis.

[16] Ferreira V.H. (2022). Homotypic and heterotypic immune responses to Omicron variant in immunocompromised patients in diverse clinical settings. Nat Commun.

[17] Assawakosri S. (2022). Neutralizing activities against the omicron variant after a heterologous booster in healthy adults receiving two doses of CoronaVac vaccination. J Infect Dis.

[18] Pulvirenti F. (2021). B cell response induced by SARS-CoV-2 infection is boosted by the BNT162b2 vaccine in primary antibody deficiencies. Cells.

[19] Arroyo-Sánchez D. (2022). Immunogenicity of anti-SARS-CoV-2 vaccines in common variable immunodeficiency. J Clin Immunol.

[20] Thümmler L. (2022). Cellular and humoral immunity after the third vaccination against SARS-CoV-2 in hematopoietic stem-cell transplant recipients. Vaccines (Basel).

[21] Tang K., Wei Z., Wu X. (2022). Impaired serological response to COVID-19 vaccination following anticancer therapy: a systematic review and meta-analysis. J Med Virol.

[22] Mrak D. (2022). Immunogenicity and safety of a fourth COVID-19 vaccination in rituximab-treated patients: an open-label extension study. Ann Rheum Dis.

[23] Lehrnbecher T. (2022). Longitudinal immune response to 3 doses of messenger RNA vaccine against coronavirus disease 2019 (COVID-19) in pediatric patients receiving chemotherapy for cancer. Clin Infect Dis.

[24] Azzolini E. (2022). mRNA COVID-19 vaccine booster fosters B- and T-cell responses in immunocompromised patients. Life Sci Alliance.

[25] Dayam R.M. (2022). Accelerated waning of immunity to SARS-CoV-2 mRNA vaccines in patients with immune-mediated inflammatory diseases. JCI Insight.

[26] Sand IK et al. Evaluation of immunological responses to third COVID-19 vaccine among people treated with sphingosine receptor-1 modulators and anti-CD20 therapy. medRxiv; 2022. 10.1016/j.msard.2022.104486 PMC9794520 36628884

[27] Hadjadj J. (2022). Immunogenicity of BNT162b2 vaccine against the Alpha and Delta variants in immunocompromised patients with systemic inflammatory diseases. Ann Rheum Dis.

[28] Simon B. (2022). SARS-CoV-2 antibody and T cell response after a third vaccine dose in hemodialysis patients compared with healthy controls. Vaccines.

[29] Karakizlis H. (2022). Immunogenicity and reactogenicity of homologous mRNA-based and vector-based SARS-CoV-2 vaccine regimens in patients receiving maintenance dialysis. Clin Immunol.

[30] Tallantyre E.C. (2022). Response to COVID-19 booster vaccinations in seronegative people with multiple sclerosis. Mult Scler Relat Disord.

[31] Peled Y. (2022). Kinetics of cellular and humoral responses to third BNT162B2 COVID-19 vaccine over six months in heart transplant recipients – implications for the omicron variant. J Heart Lung Transplant.

[32] Tobudic S. (2022). Immune response after mRNA COVID-19 vaccination in lung transplant recipients: a 6-month follow-up. Vaccines (Basel).

[33] Corradini P. (2022). Humoral and T-cell immune response after three doses of mRNA SARS-CoV-2 vaccines in fragile patients: the Italian VAX4FRAIL study. Clin Infect Dis.

[34] Davidov Y. (2022). A third dose of the BNT162b2 mRNA vaccine significantly improves immune responses among liver transplant recipients. J Hepatol.

[35] Charmetant X. (2022). Infection or a third dose of mRNA vaccine elicits neutralizing antibody responses against SARS-CoV-2 in kidney transplant recipients. Sci Transl Med.

[36] Stumpf J. (2022). Anti-SARS-CoV-2 revaccination success in kidney transplant recipients with no initial humoral response is linked to primary vaccine type. Front Med (Lausanne).

[37] Jotschke S. (2022). Longitudinal humoral and cellular immune responses following SARS-CoV-2 vaccination in patients with myeloid and lymphoid neoplasms compared to a reference cohort: results of a prospective trial of the East German study group for hematology and oncology (OSHO). Cancers.

[38] Ministry of Health. LaW. Coronavirus (COVID-19); 2023. https://www.mhlw.go.jp/stf/seisakunitsuite/bunya/0000164708_00079.html.

[39] Kobashi Y. (2021). Seroprevalence of SARS-CoV-2 antibodies among hospital staff in rural Central Fukushima, Japan: a historical cohort study. Int Immunopharmacol.

[40] Shimazu Y. (2021). Mental distress in a clinical nurse due to a false-positive COVID-19 antibody test result during the COVID-19 epidemic in Japan: a case report. Clin Case Rep.

[41] Kobashi Y. (2022). Peak IgG antibody titers against SARS-CoV-2 spike protein following immunization with the Pfizer/BioNTech BNT162b2 vaccine. Fukushima J Med Sci.

[42] Kobashi Y. (2022). Maturing of public-private-people partnership (4P): lessons from 4P for triple disaster and subsequently COVID-19 pandemic in Fukushima. J Glob Health.

[43] Uwamino Y. (2022). Dynamics of antibody titers and cellular immunity among Japanese healthcare workers during the 6 months after receiving two doses of BNT162b2 mRNA vaccine. Vaccine.

[44] Arbel R. (2022). Effectiveness of a second BNT162b2 booster vaccine against hospitalization and death from COVID-19 in adults aged over 60 years. Nat Med.

[45] Lee C.M. (2023). Low humoral and cellular immune responses early after breakthrough infection may contribute to severe COVID-19. Front Immunol.

[46] Petrilli C.M. (2020). Factors associated with hospital admission and critical illness among 5279 people with coronavirus disease 2019 in New York City: prospective cohort study. BMJ.

[47] Jung S. (2022). The generation of stem cell-like memory cells early after BNT162b2 vaccination is associated with durability of memory CD8+ T cell responses. Cell Rep.

[48] Murray C.E. (2023). Cellular and humoral immunogenicity of the COVID-19 vaccine and COVID-19 disease severity in individuals with immunodeficiency. Front Immunol.

