# Supplementary material for: Kinetics of humoral and cellular immune responses 5 months post-COVID-19 booster dose by immune response groups at the peak immunity phase: An observational historical cohort study using the Fukushima vaccination community survey
Source: Vaccine X. 2024 Sep 12;20:100553. doi: 10.1016/j.jvacx.2024.100553 (PMC11416657; doi:10.1016/j.jvacx.2024.100553)
Supplement: Supplementary Data 1 [file mmc1.docx]

Supplementary Material

Yurie Kobashi,^1,2^ Takeshi Kawamura,^3,4^ Yuzo Shimazu,^1^ Yudai Kaneko,^4,5^ Yoshitaka Nishikawa,^2^ Akira Sugiyama,^3^ Yuta Tani,^6^ Aya Nakayama,^3^ Makoto Yoshida,^6^ Zho Tianchen,^1^ Chika Yamamoto,^1^ Hiroaki Saito,^1^ Morihito Takita,^1^ Masatoshi Wakui,^7^ Tatsuhiko Kodama,^4^ and Masaharu Tsubokura^1,2^

*** Correspondence:** Masaharu Tsubokura
tsubo-m@fmu.ac.jp

# Supplementary Figures and Tables

## Supplementary Figures

**A**

**B**

**C**

**Supplementary Figure 1.** Distribution of the neutralizing activity (Nab), IgG antibody titers, and ELISpot at T1(March 2022) and T2 (June 2022)

## Supplementary Tables

**Supplementary Table 1. Baseline characteristics of the different immune groups at T1 (N = 983)**

|  | Group 1 | Group 2 | Group 3 | Group 4 | p-value |
| --- | --- | --- | --- | --- | --- |
| Age, years (median [IQR]) | 52 [39–64] | 54.5 [44–66] | 67 [56–77] | 66 [51–81] | <0.001 |
| Sex: female, n (%) | 404 (66.8) | 124 (77.5) | 82 (61.7) | 49 (57.7) | 0.004 |
| Day from 3rd vaccine (median [IQR]) | 142 [122–157] | 155 [141–166] | 146 [122–157] | 151 [137–159] | <0.001 |
| Interval between 1st and 2nd vaccine (median [IQR]) | 21 [21–21] | 21 [21–21] | 21 [21–21] | 21 [21–21] | 0.117 |
| Interval between 2ndt and 3rd vaccine (median [IQR]) | 230 [217–239] | 231 [217–234] | 231 [222–242] | 231 [219–237] | 0.38 |
| Vaccination kind of third dose (Moderna) | 240 (39.7) | 23 (14.4) | 64 (48.1) | 29 (34.1) | <0.001 |
| Adverse reaction |  |  |  |  |  |
| Local pain (n = 978) | 394 (65.6) | 107 (67.3) | 62 (46.6) | 38 (44.7) | <0.001 |
| Whole adverse reaction (n = 974) | 429 (71.7) | 100 (62.9) | 50 (37.6) | 27 (32.1) | <0.001 |
| Smoking habit (n = 964) | 112 (18.7) | 30 (19.6) | 21 (16.2) | 11 (13.4) | 0.59 |
| Alcohol consumption (n = 952) | 245 (41.3) | 56 (37.3) | 45 (34.9) | 33 (41.3) | 0.51 |
| Daily medicine |  |  |  |  |  |
| Steroid (n = 961) | 11 (1.9) | 5 (3.2) | 5 (3.8) | 5 (6.0) | 0.121 |
| Immunosuppression (n = 960) | 5 (0.9) | 4 (2.6) | 3 (2.3) | 2 (2.4) | 0.26 |
| Biologics (n = 958) | 1 (0.2) | 4 (2.6) | 0 (0.0) | 0 (0.0) | 0.002 |
| Comorbidity |  |  |  |  |  |
| Hypertension (n = 981) | 135 (22.4) | 37 (23.1) | 50 (37.6) | 36 (42.9) | 0.005 |
| Diabetes (n = 981) | 40 (6.6) | 8 (5.0) | 17 (12.8) | 13 (15.5) | 0.003 |
| Dyslipidemia (n = 981) | 51 (8.4) | 15 (9.4) | 11 (8.3) | 10 (11.9) | 0.75 |
| BMI over 25 (n = 867) | 180 (33.8) | 31 (21.0) | 32 (29.1) | 15 (19.7) | 0.004 |

Chi square test was employed for categorical variables, and ANOVA was employed for continuous variables.

BMI, body mass index; IQR, interquartile range

**Supplementary Table 2. Number of participants in different age groups in all immune groups at T1 (N = 983)**

| Age | Group 1 | Group 2 | Group 3 | Group 4 |
| --- | --- | --- | --- | --- |
| ≤39 years | 158 | 21 | 9 | 7 |
| 40–59 years | 248 | 74 | 33 | 20 |
| 60–79 years | 161 | 51 | 61 | 35 |
| ≥80 years | 38 | 14 | 30 | 23 |

**Supplementary Table 3. Logistic regression analysis for lower 1/3 group at T2 (June 2022) each assay (n = 803)**

|  | Dependent variable:  IgG (S) lower 1/3 | |  | Dependent variable:  T-spot (S) lower 1/3 | |  | Dependent variable:  Lower 1/3 IgG (S)  and 1/3 T-spot (S) | |
| --- | --- | --- | --- | --- | --- | --- | --- | --- |
|  | OR (95% CI) | *p* value |  | OR (95% CI) | *p* value |  | OR (95% CI) | p-value |
| Age (years) | 1.02 (1.01–1.03) | **0.004** |  | 1.03 (1.02–1.05) | **<0.001** |  | 1.05 (1.03–1.06) | **<0.001** |
| Sex (base: male) | 1.30 (0.89–1.89) | 0.176 |  | 0.78 (0.54–1.12) | 0.177 |  | 0.96 (0.60–1.54) | 0.865 |
| Day from 4th vaccine (days) | 1.01 (1.00–1.02) | **0.004** |  | 1.00 (0.99–1.01) | 0.941 |  | 1.00 (0.99–1.02) | 0.453 |
| Type of third dose vaccination  (base: BNT162b2) | 0.48 (0.31–0.73) | **0.001** |  | 0.89 (0.60–1.33) | 0.578 |  | 0.52 (0.31–0.88) | **0.015** |
| Whole adverse reaction | 0.49 (0.35–0.70) | **<0.001** |  | 0.42 (0.30–0.60) | **<0.001** |  | 0.41 (0.26–0.64) | **<0.001** |
| Smoking habit | 1.95 (1.26–3.00) | **0.003** |  | 1.57 (1.02–2.40) | **0.039** |  | 1.78 (1.02–3.10) | **0.042** |
| Alcohol consumption | 0.84 (0.59–1.18) | 0.313 |  | 0.53 (0.37–0.75) | **<0.001** |  | 0.73 (0.46–1.15) | 0.178 |
| Daily medicine |  |  |  |  |  |  |  |  |
| Steroids | 0.94 (0.34–2.65) | 0.912 |  | 0.44 (0.14–1.39) | 0.164 |  | 0.82 (0.25–2.67) | 0.745 |
| Immunosuppression | 2.58 (0.50–13.41) | 0.259 |  | 40.67 (3.43–482.05) | **0.003** |  | 8.73 (1.60–47.62) | **0.012** |
| Biologics | 11.52 (0.96–138.05) | 0.054 |  | 0.32 (0.01–7.73) | 0.486 |  | 2.00 (0.15–26.20) | 0.598 |
| Comorbidity |  |  |  |  |  |  |  |  |
| Hypertension | 1.21 (0.81–1.81) | 0.356 |  | 0.83 (0.56–1.25) | 0.383 |  | 0.81 (0.50–1.32) | 0.397 |
| Diabetes | 1.44 (0.80–2.60) | 0.221 |  | 1.01 (0.56–1.84) | 0.964 |  | 1.16 (0.58–2.30) | 0.672 |
| Dyslipidemia | 1.01 (0.60–1.72) | 0.959 |  | 0.77 (0.45–1.31) | 0.330 |  | 0.96 (0.51–1.84) | 0.912 |
| BMI over 25 | 0.63 (0.43–0.91) | **0.014** |  | 0.92 (0.64–1.32) | 0.652 |  | 0.89 (0.55–1.42) | 0.623 |

BMI, body mass index
